# Effects of Light and Daytime on the Regulation of Chitosan-Induced Stomatal Responses and Defence in Tomato Plants

**DOI:** 10.3390/plants9010059

**Published:** 2020-01-02

**Authors:** Zalán Czékus, Péter Poór, Irma Tari, Attila Ördög

**Affiliations:** 1 Department of Plant Biology, University of Szeged, H-6726 Szeged, Közép fasor 52., Hungary; czekus.z@bio.u-szeged.hu (Z.C.); tari@bio.u-szeged.hu (I.T.); aordog@bio.u-szeged.hu (A.Ö.); 2 Doctoral School of Biology, University of Szeged, H-6726 Szeged, Közép fasor 52., Hungary

**Keywords:** chitosan, chlorophyll *a* fluorescence, nitric oxide, reactive oxygen species, stomata

## Abstract

Closure of stomata upon pathogenesis is among the earliest plant immune responses. However, our knowledge is very limited about the dependency of plant defence responses to chitosan (CHT) on external factors (e.g., time of the day, presence, or absence of light) in intact plants. CHT induced stomatal closure before dark/light transition in leaves treated at 17:00 hrs and stomata were closed at 09:00 hrs in plants treated at dawn and in the morning. CHT was able to induce generation of reactive oxygen species (ROS) in guard cells in the first part of the light phase, but significant nitric oxide production was observable only at 15:00 hrs. The actual quantum yield of PSII electron transport (Φ_PSII_) decreased upon CHT treatments at 09:00 hrs in guard cells but it declined only at dawn in mesophyll cells after the treatment at 17:00 hrs. Expression of *Pathogenesis-related 1* (*PR1*) and *Ethylene Response Factor 1* were already increased at dawn in the CHT-treated leaves but *PR1* expression was inhibited in the dark. CHT-induced systemic response was also observed in the distal leaves of CHT-treated ones. Our results suggest a delayed and daytime-dependent defence response of tomato plants after CHT treatment at night and under darkness.

## 1. Introduction

Environmental conditions, such as light, humidity, and temperature can crucially influence plant immunity and infection [1,2]. Especially, light-induced signalling, the regulation by circadian clock or the photosynthetic activity can influence physiological, biochemical, and molecular responses of plants [3,4,5]. Immune strategies of plants also show time-of-day-dependency mediated by several phytohormones, such as salicylic acid (SA), jasmonic acid (JA), and ethylene (ET) [5,6,7]. 

Stomata, the microscopic pores formed by a pair of guard cells in the epidermis play role not only in the transpiration and exchange of gases but also provide natural entry points for various pathogens into the leaves. Closure of stomata in response to pathogens is one of the earliest event among plant immune responses [8,9]. Pattern recognition receptors (PRR) are able to recognize a wide variety of plant pathogens by detecting microbial- or pathogen-associated molecular patterns (MAMPs or PAMPs) and induce rapid stomatal closure as a part of pattern-triggered immunity (PTI) [10,11]. Circadian-regulated JA and SA synthesis, as well as the production of reactive oxygen- (ROS) and nitrogen species (RNS) are important components of guard cell signalling during the infection [12,13]. It was found that the transcription factors CIRCADIAN CLOCK ASSOCIATED1 (CCA1) and its close homolog LATE ELONGATED HYPOCOTYL (LHY), are able to directly affect the abundance of GLYCINE-RICH RNA BINDING PROTEIN 7 (GRP7) via binding to its promoter at different times of the day, which in turn regulates stomatal aperture and thereby stomatal defence against *Pseudomonas syringae* in *Arabidopsis thaliana* [14]. Another component involved in the clock-regulated stomata-based pre-invasive defence is the time for coffee (TIC), a night-expressed clock gene. TIC inhibits JA signalling in the evening and contributes to a stronger JA responsiveness in the morning [15,16]. Thus, both the daytime and/or the presence or absence of light seem to be crucial factor in the phytohormone-mediated response of plants against various pathogens. 

It can be concluded that diurnal effects of light can significantly influence plant-pathogen interactions. It is well known that light can directly inhibit spore germination, germ tube growth, and reduces the infection efficiency of many pathogenic fungi, such as *Botrytis cinerea* [17], *Fusarium graminearum* [18], *Phakopsora pachyrhizi* [19], and *Puccinia hemerocallidis* [20]. Thus, plants can be subjected to a greater challenge by many fungal pathogens at night than during the day [3]. Although stomata are closed at night and provide the first line of defence in the dark, at dawn stomata start to open thus provide infection window for many pathogens [21]. Decreased susceptibility to *Botrytis cinerea* was observed in *Arabidopsis thaliana* after inoculation at dawn compared with the night [22]. Besides rapid production of ROS and RNS at the infection sites, the initiation of the biosynthesis of SA and JA and increased expression of pathogenesis-related (*PR*) genes were also induced, which resulted in the hypersensitive response (HR). The localized contact with pathogens can further activate systemic acquired resistance (SAR) even at the whole plant level, which can protect the infected plant against the subsequent pathogen attack [23,24,25]. Before, after, or during the infection the quality and the quantity of the light can influence the development of HR and SAR in the leaves [7,26,27,28]. It was observed that SAR development (regulated by SA, methyl salicylate (MeSA), and JA) was more significant following the pathogen infection in the morning compared to the infection in the late afternoon followed by the dark period [29]. Thus, understanding the role of phytohormones in plant responses to pathogen infection in presence or absence of light, or in different daytimes is an important perspective for future plant stress physiology and plant protection studies.

Fungal pathogen attack can be mimicked by elicitor molecules such as chitosan (β-1,4-linked D-glucosamine; CHT), which is the deacylated derivative of the fungal cell wall component chitin [30]. It was found in stomata assay that CHT inhibits light-induced stomatal opening in tomato, which is mediated by Ca^2+^ and H_2_O_2_ [31,32]. It was also observed that not only a rapid ROS, but also high RNS production was induced by CHT before the initiation of stomatal closure in epidermal peels [33]. Interestingly, either endogenous abscisic acid (ABA) or endogenous JA was not involved in CHT-induced stomatal closure in *Arabidopsis thaliana* [34]. Furthermore, SA seems to be the key component in CHT-induced stomatal closure based on the investigation of epidermal peels of SA-deficient *nahG* mutants [35]. As defence mechanisms are based mainly on hormonal interactions, the study of the daytime-dependency of occurrence of defence hormones and the expression of hormonal response genes are very important. Moreover, it was shown that CHT reduced the photosynthetic linear electron transport rate (ETR) and ion channel activity in the guard cells of *Vicia faba*, thus contributed to the induction of stomatal closure [36]. It is well known that chloroplast function (biosynthesis of SA, JA and ABA; production of energy and metabolic precursors) is required during different plant-pathogen interactions [3]. It is also known that guard cell chloroplasts are responsible (at least in part) for the stimulation of stomatal opening, for ATP supply, for blue-light signalling and production of osmolytes [37]. However, the daytime-dependent role of chloroplasts in plant responses to pathogens is less known. In addition, the interaction or self-regulation of the photosynthetic activity of guard- and mesophyll cells under the infection is also poorly understood [1].

In this work, the daytime- and light-dependent effects of CHT was investigated the first time in intact leaves of tomato plants on the stomatal movements, ROS and NO production in guard cells. Changes in the photosynthetic activity of guard- and mesophyll-cells were also compared in CHT-treated and untreated distal leaves of intact tomato plants. Our experiments were also focused on whether CHT treatments could mediate stomatal closure and/or SAR in untreated distal leaves of intact plants. The development of SAR was investigated in these distal leaves based on the analysis of the expression profile of defence genes mediated by SA and JA/ET.

## 2. Results

**Figure 1:**
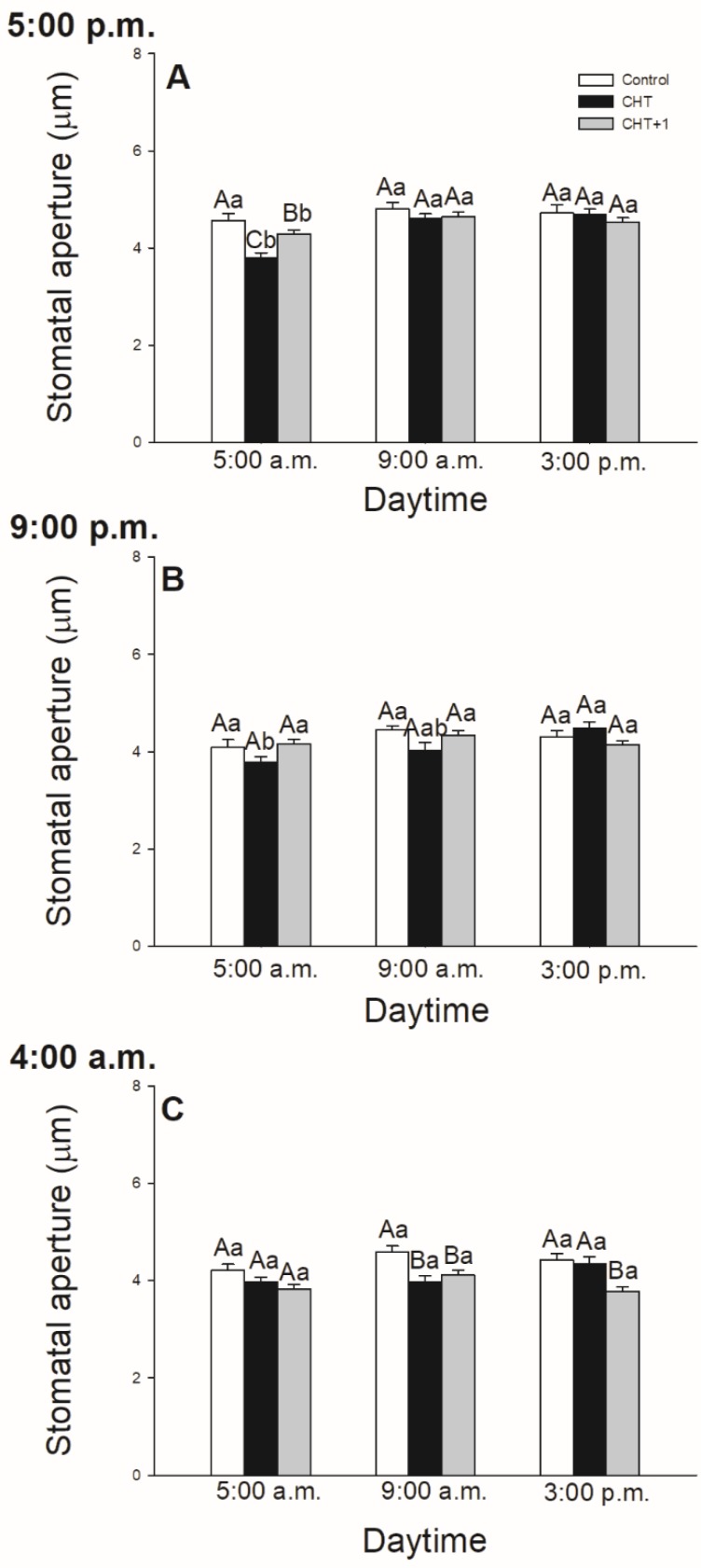
Changes in the size of stomatal apertures on the abaxial epidermal strips of intact tomato plants treated foliar with 100 μg mL^-1^ chitosan (CHT) dissolved in the experimental buffer (1 mM AA, 10 mM MES/TRIS, 10 mM KCl, 0.1 mM CaCl_2_, pH 6.15) at different daytimes: at the end of light cycle (17:00 hrs) (**A**)), at night (21:00 hrs; (**B**)), and at dawn (04:00 hrs; (**C**)). Measurements were disposed at constant times after treatments (17:00, 09:00, and 15:00 hrs). Means ± SE, n = 3. Means were analyzed by two-way ANOVA, significant differences among the data were analyzed by Holm–Sidak test. Mean values significantly different at *P* < 0.05 were signed with different letters, upper case letters indicate the effects of the treatment at the same daytime, and lower case letters indicate the effects of the daytime under the same treatment. (Control: treatment with 1 mM Acetic acid buffer (AA); CHT: treatment with 100 μg mL^-1^ chitosan in experimental buffer containing AA; CHT+1: untreated distal leaf level from the CHT-treated one).

**Figure 2:**
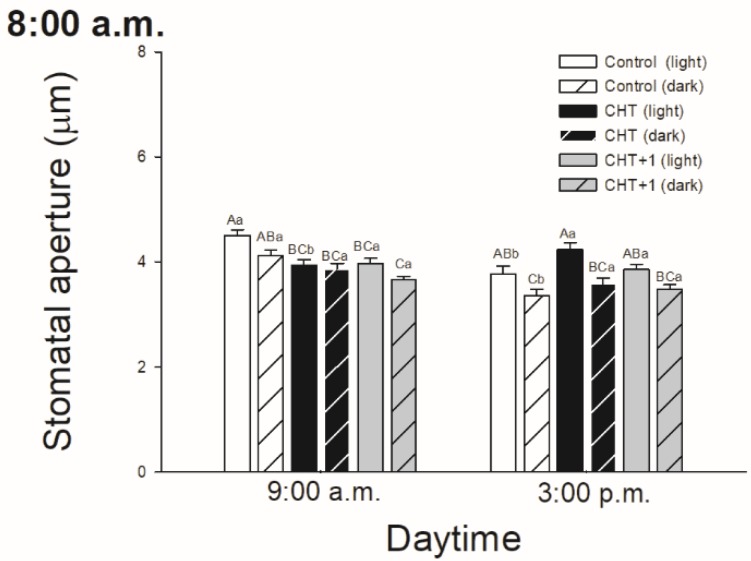
Changes in the size of stomatal apertures on the abaxial epidermal strips of intact tomato plants treated foliar with 100 μg mL^-1^ chitosan (CHT) dissolved in the experimental buffer (1 mM AA, 10 mM MES/TRIS, 10 mM KCl, 0.1 mM CaCl_2_, pH 6.15) in the morning at 08:00 hrs, keeping under light (normal columns) or in continuous darkness (stripped columns). Measurements were disposed at constant times after treatments (09:00 hrs and 15:00 hrs). Means ± SE, n = 3. Means were analyzed by two-way ANOVA, significant differences among the data were analyzed by Holm–Sidak test. Mean values significantly different at *P* < 0.05 were signed with different letters, upper case letters indicate the effects of the treatment at the same daytime, and lower case letters indicate the effects of the daytime under the same treatment. (Control: treatment with 1 mM Acetic acid buffer (AA); CHT: treatment with 100 μg mL^-1^ chitosan in experimental buffer containing AA; CHT+1: untreated distal leaf level from the CHT-treated one).

In intact tomato leaves, stomata started to open at dawn and reached the maxima of stomatal pore size around 12 h then started to close during the afternoon (results not shown). Interestingly, CHT induced stomatal closure before dark/light transition only in the leaves treated at 17:00 hrs. (Figure 1A), but it also caused stomatal closure at 09:00 hrs in both types of the treatments (in the light and in artificial dark) in the plants treated at dawn and in the morning during sampling (Figure 1C; Figure 2). In contrast to this, the size of stomatal aperture in the plants treated in the evening did not change significantly in samples collected at 17:00 hrs and 09:00 hrs. (Figure 1B). Moreover, our results showed that CHT did not affect stomatal movements in the late afternoon (sampling at 15:00 hrs) compared to the control independently of the time of the treatment (Figure 1). 

At the same time, CHT treatments induced significant stomatal closure in the distal leaves from CHT-treated ones (on the 5th leaf levels of intact tomato plant) 1 hour later after the elicitor treatment in the morning (Figure 2).

**Figure 3:**
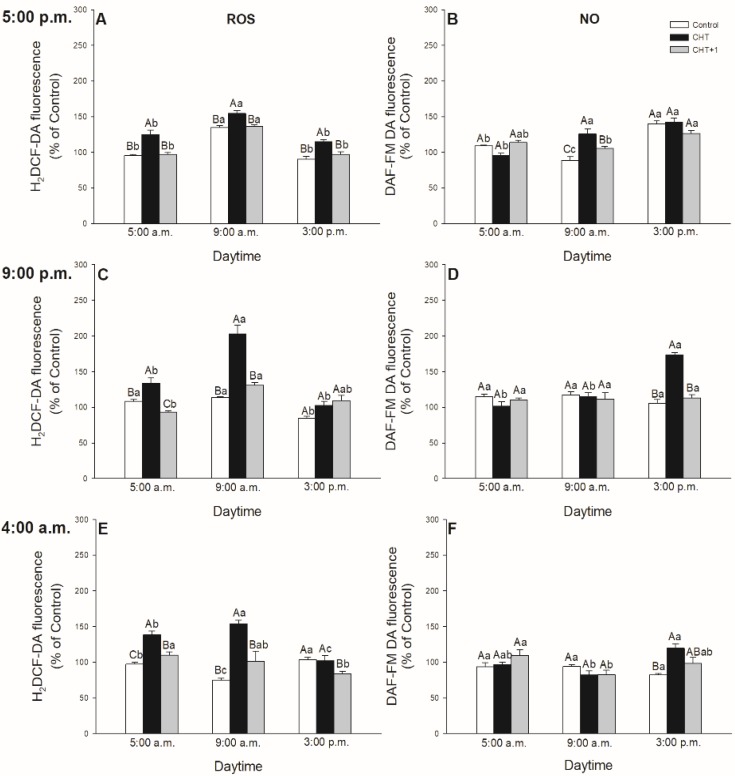
Changes in the production of ROS (**A**,**C**,**E**) and NO (**B**,**D**,**F**) in stomata on the abaxial epidermal strips of intact tomato plants treated foliar with 100 μg mL^-1^ chitosan (CHT) dissolved in the experimental buffer (1 mM AA, 10 mM MES/TRIS, 10 mM KCl, 0.1 mM CaCl_2_, pH 6.15) at different daytimes: at the end of light cycle (17:00 hrs; (A)), at night (21:00 hrs; (B)), at dawn (04:00 hrs; (C)). Measurements were disposed at constant times after treatments (5:00 a.m., 9:00 a.m. and 3:00 p.m.). Means ± SE, n = 3. Means were analyzed by two-way ANOVA, significant differences among the data were analyzed by Holm–Sidak test. Mean values significantly different at *P* < 0.05 were signed with different letters, upper case letters indicate the effects of the treatment at the same daytime, and lower case letters indicate the effects of the daytime under the same treatment. (Control: treatment with 1 mM Acetic acid buffer (AA); CHT: treatment with 100 μg mL^-1^ chitosan in experimental buffer containing AA; CHT+1: untreated distal leaf level from the CHT-treated one).

As stomatal movements are regulated by ROS and NO, the possible daytime effects on CHT-induced production of ROS and NO in guard cells were examined in parallel with the changes in the stomatal aperture size. In all cases, CHT induced significant ROS generation in guard cells in the first part of the light phase of the day independently of the daytime of CHT treatments (Figure 3). However, ROS burst was significantly higher at 09:00 compared to at 05:00 hrs, and especially compared to the leaves treated in the evening (at 21:00 hrs) (Figure 3C). 

**Figure 4:**
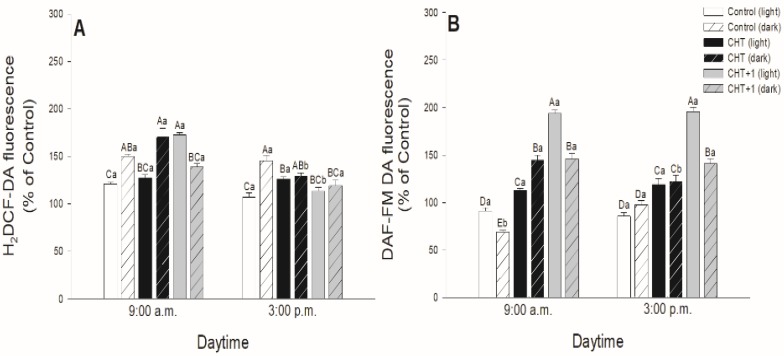
Changes in the production of ROS (A) and NO (B) in stoma on the abaxial epidermal strips of intact tomato plants treated foliar with 100 μg mL^-1^ chitosan (CHT) dissolved in the experimental buffer (1 mM AA, 10 mM MES/TRIS, 10 mM KCl, 0.1 mM CaCl_2_, pH 6.15) in the morning, keeping under light (normal columns) or in continuous darkness (stripped columns)]. Measurements were disposed at constant times after treatments (09:00 and 15:00 hrs). Means ± SE, n = 3. Means were analyzed by two-way ANOVA, significant differences among the data were analyzed by Holm–Sidak test. Mean values significantly different at *P* < 0.05 were signed with different letters, upper case letters indicate the effects of the treatment at the same daytime, and lower case letters indicate the effects of the daytime under the same treatment. (Control: treatment with 1 mM Acetic acid buffer (AA); CHT: treatment with 100 μg mL^-1^ chitosan in experimental buffer containing AA; CHT+1: untreated distal leaf level from the CHT-treated one).

Higher ROS production was also observed in CHT-treated stomata under darkness at 09:00 hrs and in the distal leaves from CHT-treated ones (Figure 4A). Interestingly, CHT promoted significant NO generation in the afternoon in case of the treatments in the dark period of the day (sampling at 15:00 hrs, treatments at 21:00 and 04:00 hrs; Figure 3D,F) and upon the combined CHT and artificial dark treatments (Figure 4B). NO production was significant at 09:00 hrs upon CHT treatment at 17:00 hrs (Figure 3A). Surprisingly, NO levels elevated also in the distal leaves from the CHT-treated ones in the samples treated in the morning (09:00 hrs) (Figure 4B).

**Figure 5:**
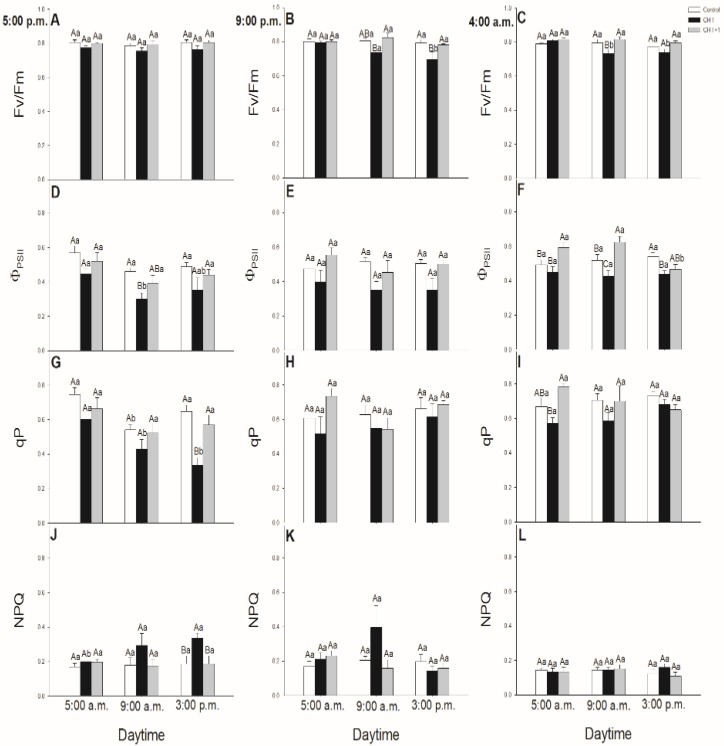
Changes in the chlorophyll *a* fluorescence parameters [Fv/Fm (**A–C**), Φ_PSII_ (**D–F**), qP (**G–I**), NPQ (**J–L**)] of stomata on the abaxial epidermal strips of intact tomato plants treated foliar with 100 μg mL^–^^1^ chitosan (CHT) dissolved in the experimental buffer (1 mM AA, 10 mM MES/TRIS, 10 mM KCl, 0.1 mM CaCl_2_, pH 6.15) at different daytimes: at the end of light cycle (17:00 hrs), at night (21:00 hrs), at dawn (04:00 hrs). Measurements were disposed at constant times after treatments (05:00, 09:00 and 15:00 hrs). Means ± SE, n = 3. Means were analyzed by two-way ANOVA, significant differences among the data were analyzed by Holm–Sidak test. Mean values significantly different at *P* < 0.05 were signed with different letters, upper case letters indicate the effects of the treatment at the same daytime, and lower case letters indicate the effects of the daytime under the same treatment. (Control: treatment with 1 mM Acetic acid buffer (AA); CHT: treatment with 100 μg mL^–^^1^ chitosan in experimental buffer containing AA; CHT+1: untreated distal leaf level from the CHT-treated one).

The daytime- and light-dependence of the PSII activity was investigated after CHT treatments in order to ascertain the possible effects of CHT mediated by light and the circadian rhythm on the PSII activity. The maximal quantum yield of PSII photochemistry (F_v_/F_m_) decreased slightly at 09:00 hrs upon CHT treatments, mostly in the guard cells treated in the dark (Figure 5C). In addition, the actual quantum yield of PSII electron transport in the light-adapted state (Φ_PSII_) also decreased upon CHT treatment at dawn (Figure 5F). The photochemical quenching coefficient (qP) significantly decreased at 15:00 hrs in the leaves treated in the afternoon with CHT (Figure 5G). 

**Figure 6:**
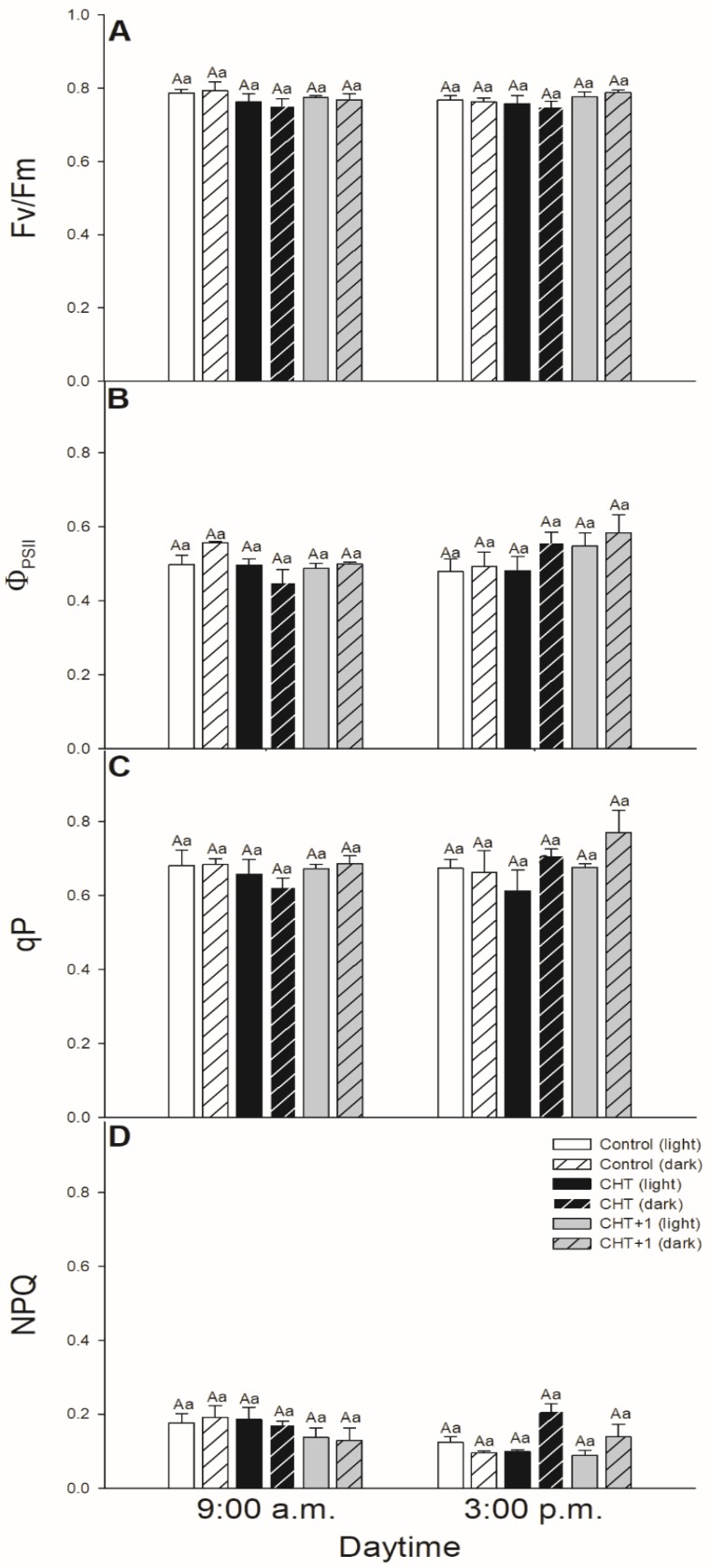
Changes in the chlorophyll *a* fluorescence parameters [Fv/Fm (**A**), Φ_PSII_ (**B**), qP (**C**), NPQ (**D**)] of stomata on the abaxial epidermal strips of intact tomato plants treated foliar with 100 μg mL^–^^1^ chitosan (CHT) dissolved in the experimental buffer (1 mM AA, 10 mM MES/TRIS, 10 mM KCl, 0.1 mM CaCl_2_, pH 6.15) in the morning: 08:00 hrs, keeping under light (normal columns) or in continuous darkness (stripped columns)]. Measurements were disposed at constant times after treatments (09:00 and 15:00 hrs). Means ± SE, n = 3. Means were analyzed by two-way ANOVA, significant differences among the data were analyzed by Holm-Sidak test. Mean values significantly different at *P* < 0.05 were signed with different letters, upper case letters indicate the effects of the treatment at the same daytime, and lower case letters indicate the effects of the daytime under the same treatment. (Control: treatment with 1 mM Acetic acid buffer (AA); CHT: treatment with 100 μg mL^–^^1^ chitosan in experimental buffer containing AA; CHT+1: untreated distal leaf level from the CHT-treated one).

Interestingly, when leaves were treated with CHT in the morning at 08:00 hrs, when stomata were already opened, Fv/Fm, Φ_PSII_ and qP did not change significantly in the guard cells (Figure 6A–C). Moreover, CHT did not influence significantly the non-photochemical quenching (NPQ) parameter in stomata (Figure 5J–L; Figure 6D) except of the case when leaves were treated at 17:00 hrs and measured at 15:00 hrs in the next day (Figure 5J). Furthermore, we were not able to measure any significant decrease in the chlorophyll *a* fluorescence parameters in the stomata of distal leaves from the CHT-treated ones in the first light period after the fungal elicitor application.

**Figure 7:**
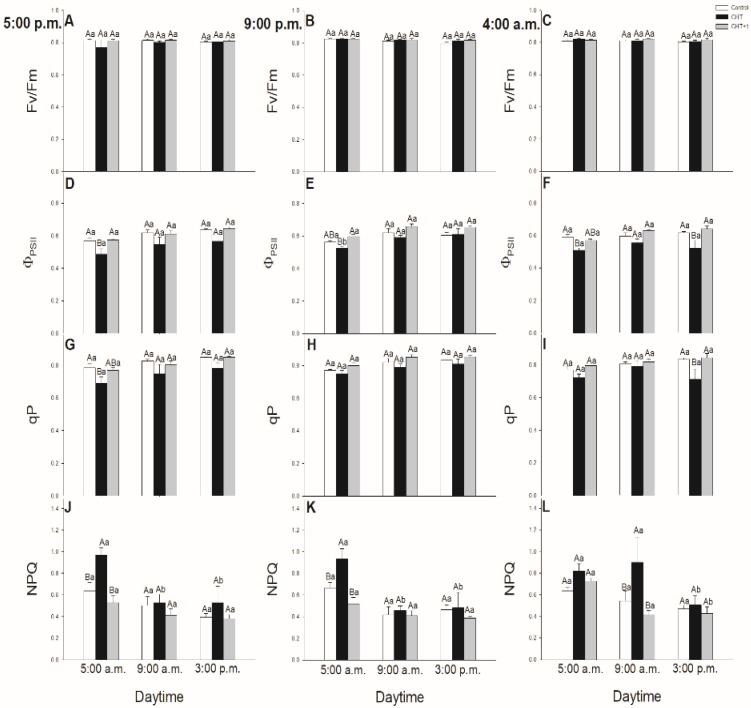
Changes in the chlorophyll *a* fluorescence parameters Fv/Fm (**A**–**C**), Φ_PSII_ (**D**–**F**), qP (**G**–**I**), NPQ (**J**–**L**) of leaves of intact tomato plants treated foliar with 100 μg mL^–^^1^ chitosan (CHT) dissolved in the experimental buffer (1 mM AA, 10 mM MES/TRIS, 10 mM KCl, 0.1 mM CaCl_2_, pH 6.15) at different daytimes: at the end of light cycle (17:00 hrs), at night (21:00 hrs), at dawn (04:00 hrs). Measurements were disposed at constant times after treatments (05:00, 09:00, and 15:00 hrs). Means ± SE, n = 3. Means were analyzed by two-way ANOVA, significant differences among the data were analyzed by Holm–Sidak test. Mean values significantly different at *P* < 0.05 were signed with different letters, upper case letters indicate the effects of the treatment at the same daytime, and lower case letters indicate the effects of the daytime under the same treatment. (Control: treatment with 1 mM Acetic acid buffer (AA); CHT: treatment with 100 μg mL^–^^1^ chitosan in experimental buffer containing AA; CHT+1: untreated distal leaf level from the CHT-treated one).

To assess whether the foliar application of CHT on intact plants results in changes in the chlorophyll *a* fluorescence parameters of the mesophyll cells, photosynthetic activity measurements were carried out in the leaves after CHT treatments applied at different daytimes. Parameters of Φ_PSII_ and qP were significantly decreased at dawn in case of the leaves treated with CHT at 17:00 hrs (Figure 7D,G) and in parallel NPQ significantly increased at this time point (Figure 7J). 

**Figure 8:**
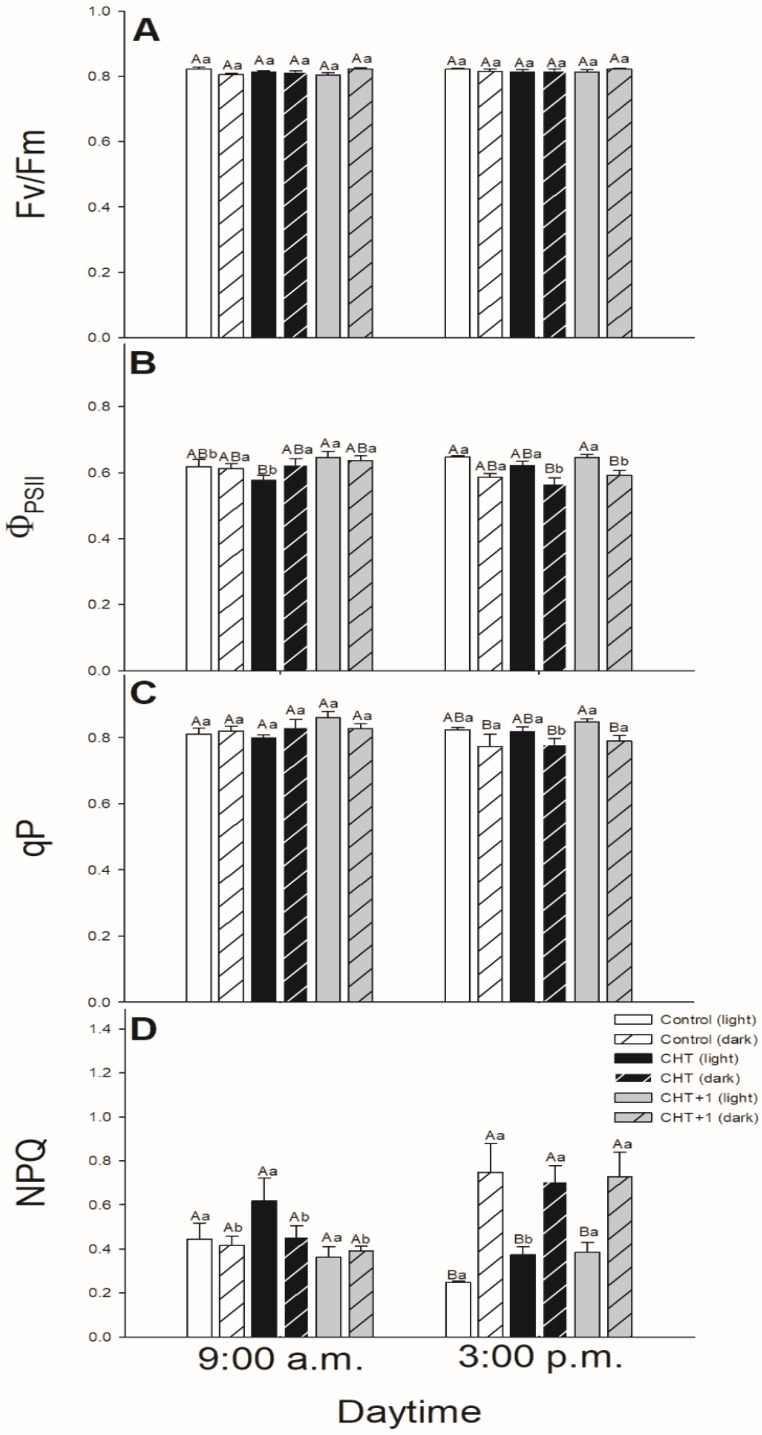
Changes in the chlorophyll *a* fluorescence parameters [Fv/Fm (**A**), Φ_PSII_ (**B**), qP (**C**), NPQ (**D**)] of leaves of intact tomato plants treated foliar with 100 μg mL^–^^1^ chitosan (CHT) dissolved in the experimental buffer (1 mM AA, 10 mM MES/TRIS, 10 mM KCl, 0.1 mM CaCl_2_, pH 6.15) in the morning: 08:00 hrs, keeping under light (normal columns) or in continuous darkness (stripped columns)]. Measurements were disposed at constant times after treatments (09:00 and 15:00 hrs). Means ± SE, n = 3. Means were analyzed by two-way ANOVA, significant differences among the data were analyzed by Holm–Sidak test. Mean values significantly different at *P* < 0.05 were signed with different letters, upper case letters indicate the effects of the treatment at the same daytime, and lower case letters indicate the effects of the daytime under the same treatment. (Control: treatment with 1 mM Acetic acid buffer (AA); CHT: treatment with 100 μg mL^–^^1^ chitosan in experimental buffer containing AA; CHT+1: untreated distal leaf level from the CHT-treated one).

CHT treatments were not able to induce any change in the chlorophyll *a* fluorescence parameters of the mesophyll cells in the light after treatments in the morning (Figure 8). Moreover, during the measurements of the chlorophyll *a* fluorescence parameters in the mesophyll cells of the distal leaves we were not able to detect any significant changes.

**Figure 9:**
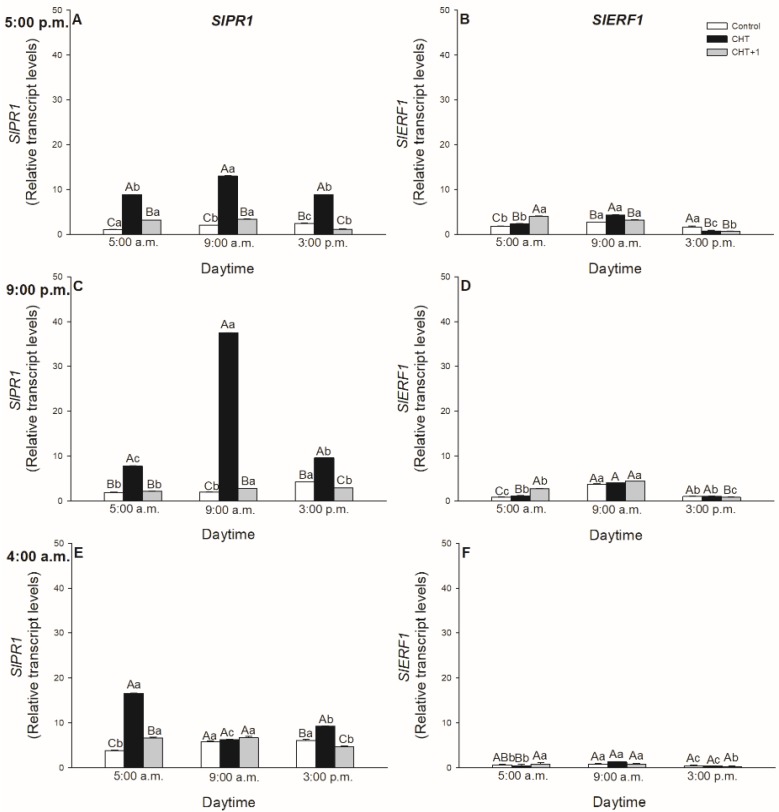
Changes in the relative expression levels of *SlPR1* (**A**,**C**,**E**) and *SlERF1* (**B**,**D**,**F**) in leaves of intact tomato plants treated foliar with 100 μg mL^–^^1^ chitosan (CHT) dissolved in the experimental buffer (1 mM AA, 10 mM MES/TRIS, 10 mM KCl, 0.1 mM CaCl_2_, pH 6.15) at different daytimes: at the end of light cycle (17:00 hrs; (**A**)), at night (21:00 hrs; (**B**)), at dawn (04:00 hrs; (**C**)). Measurements were disposed at constant times after treatments (05:00, 09:00, and 15:00 hrs). Means ± SE, n = 3. Means were analyzed by two-way ANOVA, significant differences among the data were analyzed by Holm-Sidak test. Mean values significantly different at *P* < 0.05 were signed with different letters, upper case letters indicate the effects of the treatment at the same daytime, and lower case letters indicate the effects of the daytime under the same treatment. (Control: treatment with 1 mM Acetic acid buffer (AA); CHT: treatment with 100 μg mL^–^^1^ chitosan in experimental buffer containing AA; CHT+1: untreated distal leaf level from the CHT-treated one).

**Figure 10:**
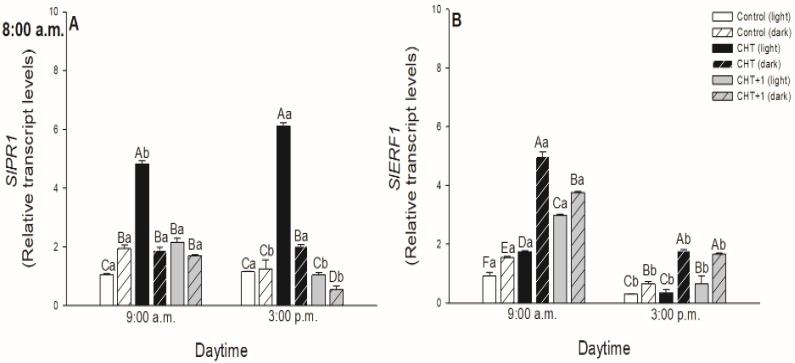
Changes in the relative expression levels of *SlPR1* (**A**) and *SlERF1* (**B**) in leaves of intact tomato plants treated foliar with 100 μg mL^-1^ chitosan (CHT) dissolved in the experimental buffer (1 mM AA, 10 mM MES/TRIS, 10 mM KCl, 0.1 mM CaCl_2_, pH 6.15) in the morning at 08:00 hrs, under light (normal columns) or in continuous darkness (stripped columns)]. Measurements were disposed at constant times after treatments (09:00 and 15:00 hrs). Means ± SE, n = 3. Means were analyzed by two-way ANOVA, significant differences among the data were analyzed by Holm-Sidak test. Mean values significantly different at *P* < 0.05 were signed with different letters, upper case letters indicate the effects of the treatment at the same daytime, and lower case letters indicate the effects of the daytime under the same treatment. (Control: treatment with 1 mM Acetic acid buffer (AA); CHT: treatment with 100 μg mL^–^^1^ chitosan in experimental buffer containing AA; CHT+1: untreated distal leaf level from the CHT-treated one).

It was also assessed, which defence hormone-responsive genes were expressed upon CHT treatment and whether the induced genes show any daytime-, light- or leaf-level-specific pattern after CHT treatments. The most significant expression was observed in the case of SA-induced *PR1* marker gene (Figure 9A,C,E; Figure 10A). Early *PR1* expression was induced already at dawn (05:00 hrs) in all the CHT-treated leaves. The highest value was observed at 09:00 hrs upon the CHT application in the early night, at 21:00 hrs. (Figure 9C). 

The most important results were observed in the morning in the early light phase, where CHT induced the *PR1* expression within 1 hour in the light (Figure 10A). The simultaneous darkening on the other hand was able to inhibit this increase in the *PR1* transcript levels, suggesting the potential light-dependent regulation of CHT-induced defence responses in tomato plants. Moreover, transcript levels of *PR1* increased also significantly after the first hour of the elicitor treatment in distal leaves from the CHT-treated ones (Figure 10A). Interestingly, the expression of *Ethylene Response Factor 1* (*ERF1*) elevated at dawn after CHT treatments at 17:00 and 21:00 hrs. (Figure 9B,D). In addition, CHT induced the expression of *ERF1* after 1 hour of the elicitor application in the morning (Figure 10B), which suggests the potential role of ET and JA in the first hours after recognition of CHT and the dominant role of SA based on *PR1* expression upon CHT in tomato plants. In contrast, *ERF1* transcript levels significantly increased under darkness, where *PR1* expression decreased in parallel suggesting the crucial role of the presence of light in the regulation of plant responses to CHT. Like transcript levels of *PR1*, *ERF1* amplified also significantly after the first hour of the elicitor treatment in distal leaves from the CHT-treated ones (Figure 10B). 

## 3. Discussion

### 3.1. Daytime and Light Dependency of Stomatal Response

In this article, the daytime- and light-dependent effects of CHT were examined in intact tomato plants. It was found that CHT caused stomatal closure at dawn, at 05:00 hrs, after the application of CHT in the previous afternoon (at 17:00 hrs) and CHT also induced stomatal closure in the morning at 09:00 hrs after the elicitor treatment at 04:00 hrs at dawn and 08:00 hrs in the early light phase. However, no significant changes were observed in case of night treatment (at 21:00 hrs) on the stomatal movement in leaves of intact plants, suggesting a daytime- or light-dependent effects on CHT-induced defence reaction. In this work, the abaxial (lower) leaf side of intact tomato leaves was used to detect the effects of fungal elicitor because stomatal density and pore area are higher at this surface influencing defence responses of plants [38]. Former studies using stomatal assay found that CHT inhibits the light-induced stomatal opening and induces stomatal closure [31,32,33]. It could be important from the aspect of defence responses of plants, therefore the time point of dark/light transition at dawn is crucial [39]. It is known that CHT treatment reduces the photosynthetic linear electron transport rate (ETR) and ion channel activity in the guard cells of *Vicia faba*, thus contributes to the induction of stomatal closure and/or inhibition of light-induced stomatal opening [36]. Moreover, CHT induces ROS and RNS production in the guard cells of epidermal peels [33] and thus stimulates stomatal closure via SA-dependent signalling [35]. Our results also demonstrated that CHT significantly caused stomatal closure before dark/light transition only if the elicitor treatment was carried out in the previous light phase of the day (at 17:00 hrs). This response of plants upon CHT treatment could be substantial, because stomata must be opened at dawn (dark/light transition) to facilitate photosynthetic CO_2_ assimilation but they provide also infection window for some pathogens [21]. Not only the dark/light transition but also the first part of the light period can influence and determine the plant defence responses in different ways. In the morning, the core circadian clock component CCA1 regulates the expression of several defence genes, such as catalases to manage ROS homeostasis and to affect defence responses through redox changes [40]. At 09:00 hrs, when the expression of defence genes shows a peak [21], stomata were closed in case of leaves treated in the morning (at 08:00 hrs) and at dawn (at 04:00 hrs). 

In contrast, in the late afternoon, the stomatal pore size of intact leaves did not differ significantly from controls after CHT treatments, which suggests different daytime-dependent signalling on whole plant level upon CHT. At this time, stomata start to close and finish to accumulate the photoassimilates [37]. This natural circadian change in the signalling may overwrite the CHT-induced stomatal movement in the afternoon. Moreover, the JA/SA hormone balance, which can determine the opening and closing of stomata is different in the afternoon where JA levels show a peak [12].

Surprisingly, based on stomatal closure a CHT-induced systemic response was detected only after one hour in the first light phase of the experiments (at 09:00) in the distal leaves of CHT-treated ones. Jelitto-Van Dooren and her co-workers were also able to observe rapid SAR development mediated by SA within two hours in distal leaves of tobacco plants followed by bacterial elicitor treatment [41]. Our results suggest firstly that the first hour after the CHT treatment could be crucial in the development of SAR, which could modify the hormonal responses in whole plant level based on the changes in the expression of defence hormone marker genes, *PR1* and *ERF1*. 

In this work, the possible daytime- and light-dependent effects on CHT-induced stomatal closure were examined firstly in parallel with the production of ROS and NO in stomata of intact leaves of tomato plants. Significant ROS production in guard cells was found in the first part of the light phase of the day independently of the daytime of CHT application. Interestingly, NO burst in the stomata was significant in the afternoon (15:00 hrs) and upon CHT-combined artificial dark treatment. These observations could be in accordance with the stomatal movements. CHT treatments promoted the inhibition of stomatal opening and/or induced stomatal closure at dawn (05:00 hrs) upon treatment in the previous afternoon (05:00 hrs) and in the early light phase (09:00 hrs) upon the treatment at dawn (04:00 hrs) and in the morning (08:00 hrs), where significantly higher ROS production was also measured in the guard cells. However, stomatal closure started in the late light phase, where NO levels were higher upon CHT treatments. It is well known that stomatal movements are controlled by several signalling molecules such as ROS and NO [42,43], which take part in phytohormone-regulated stomatal closure like in case of ABA [44,45,46], SA [47,48], JA [49], or ET [50]. It is also known that ROS and NO levels are regulated by light- and circadian rhythm [51,52]. Moreover, it has been earlier shown that the oxidative and/or nitrosative posttranslational effects of ROS and NO could also influence the function of ion channels, thus inducing stomatal closure [53]. In the case of CHT treatment, it has been reported that ROS and NO take part in the rapid stomatal closure [33]. However, the Authors used CHT-incubated abaxial epidermal peels of *Pisum sativum* for the experiments. They measured significant ROS and NO production in stomata of epidermal peels within 30 min after the CHT application [33]. Our results suggest that CHT treatments can trigger high ROS production in the guard cells at dawn and in the morning but stomatal closure is dependent on the daytime of its application in intact plants. In addition, CHT also induced significant ROS/NO production in stomata of the distal leaves from CHT-treated ones within one hour, which can contribute to induce stomatal closure in this leaf level as a part of the rapid systemic response of intact plants. 

### 3.2. Daytime and Light Dependency of Photosynthetic Activity

Chlorophyll *a* fluorescence parameters demonstrate well the effects of different stressors thus effects of CHT on PSII activity in stomata and mesophyll cells, respectively [49]. Earlier we found that CHT treatment significantly decreased the Φ_PSII_ and ETRII in *Vicia faba* guard cells [36]. In this work, the daytime- and light-dependence of the PSII activity was investigated after CHT treatments in order to ascertain the possible organ-specific effects of CHT mediated by light- and circadian rhythm in intact plants. F_v_/F_m_ decreased only slightly upon CHT treatments, but Φ_PSII_ declined after CHT treatments in the guard cells in the morning (09:00 hrs). These results suggest that CHT affects the photosynthetic performance of guard cells, which can be crucial especially in the early light phase of the day. This decline in Φ_PSII_ and the increased ROS can contribute to the stomatal closure. 

Interestingly, CHT decreased Φ_PSII_ not only in the guard cells but also in the mesophyll cells however at different daytime. CHT decreased Φ_PSII_ at dawn (05:00 hrs) in the mesophyll cells but did not change the F_v_/F_m_. Decline in Φ_PSII_ could be a part of the defence reaction when photosynthetic activation during the dark/light transition changes the chloroplastic redox homeostasis [54]. Similar to our results, Iriti et al. [55] observed that F_v_/F_m_ in CHT-treated *Phaseolus vulgaris* leaves did not differ from the control leaves after 24 h but NPQ was significantly higher upon CHT application. In case of our experiments, NPQ also increased in the CHT-treated tomato leaves at dawn. NPQ could alleviate photodamage by reducing ROS production and could improve photosynthesis at this crucial daytime [56]. These results suggest that CHT perception on the epidermis of intact leaves, which provides the first line of plant defence system, induces significant changes in the photosynthetic activity and in the signalling and metabolism of mesophyll cells in the early light phase thus can contribute to promote defence responses in leaves and plants. Besides that, surprisingly the systemic response of intact tomato plants upon CHT did not occur with a significant decrease in the chlorophyll *a* fluorescence parameters neither in the stomata nor in the mesophyll cells suggesting that photosynthetic activity is less relevant for developing the systemic response at this time point.

### 3.3. Daytime and Light Dependency of Hormonal Response Genes

The optimal timing of phytohormone-mediated defence signalling and SAR development is crucial upon pathogen infection in plants. In these processes, SA and ET/JA play a crucial role [29]. Moreover, it was also found that SA is the key molecule in CHT-induced stomatal closure instead of ABA [34]. To investigate the daytime- and light-dependent effects of CHT treatments on defence responses of tomato plants, the expression of SA- and ET/JA-responsive genes were analyzed, which has been previously showed to be active in case of bacterial pathogen *Pseudomonas syringae* in *Arabidopsis thaliana* [7,29]. Firstly, expression of the SA marker gene, *PR1* was investigated. *PR1* transcription was induced already at dawn (05:00 hrs) in the CHT-treated leaves and reached the maximum in the early light phase of the day. The highest *PR1* expression was found at 21:00 hrs in leaves treated at night suggesting a delay in defence response mediated by CHT in these plants, where stomata also did not close. 

To discriminate between circadian control and the effects of light, continuous artificial darkness was applied in the light phase at 08:00 hrs. CHT induced significant *PR1* expression within one hour in the light, but the darkness inhibited this increase in *PR1* transcript levels, suggesting the potential light-dependent regulation of CHT-induced defence responses in the elicitor-treated leaves of tomato plants. Others also found that CHT-induced *PR1* expression appears within one day, such as in rice suspension cells [57], carrot leaves [58] and in kiwifruit [59], but the daytime- and light-dependent effects of CHT application have not been investigated yet between different leaf levels in intact plants. 

The potential role of ET/JA in CHT-mediated plant responses was investigated based on the analysis of *ERF1* expression. *ERF1* encodes a transcription factor that regulates the expression of pathogen response genes that prevents disease progression and it is mediated by both ET and JA [60]. *ERF1* was also upregulated after infection with *Botrytis cinerea* and *Fusarium oxysporum*, and its induction was dependent on ET and ET plus JA signalling, respectively, whereas it was SA-independent [61]. We observed firstly that the fungal elicitor CHT induced the expression of *ERF1* at dawn showing daytime-dependent pattern upon CHT treatment at the dark/light transition. Moreover, transcript levels of *ERF1* increased significantly after one hour at 09:00 hrs in the light similarly to *PR1* expression. These results suggest that ET and JA are only responsible for the reactions occurring in the first hour after the recognition of CHT while SA has dominant role in the responses based on the higher *PR1* expression in tomato leaves in the light. In accordance with this, *ERF1* transcript levels significantly increased under darkness, where *PR1* expression decreased in parallel suggesting the crucial role of the presence of light in the regulation of plant defence responses to CHT. Furthermore, we observed significant *PR1* and *ERF1* expression in the distal leaves from CHT-treated ones only after one hour in the light, where stomatal closure and high ROS/NO production were also detected. These results provide the first evidence that, CHT can activate SAR within the first hour in the light phase of the day in intact tomato plants. These observed physiological and molecular changes can highlight the importance of the daytime and the illumination in response of intact plants to fungal elicitor CHT treatments.

## 4. Materials and methods

### 4.1. Plant Material

Tomato seeds (*Solanum lycopersicum* L. cv. Ailsa Craig) were germinated at 26 ℃ in the dark for three days, then seedlings were transferred to perlite for two weeks. Plants after that were placed in a hydroponic culture containing 2 mM Ca(NO_3_)_2_, 1 mM MgSO_4_, 0.5 mM KH_2_PO_4_, 0.5 mM Na_2_HPO_4_, 0.5 mM KCl, micronutrients (0.001 mM MnSO_4_, 0.005 mM ZnSO_4_, 0.0001 mM CuSO_4_, 0.0001 mM (NH_4_)_6_Mo_7_O_24_, 0.01 mM H_3_BO_4_), and 0.02 mM Fe(III)-EDTA at pH 5.8 as described by Poór et al. [62]. The nutrient solution was changed three times a week. Plants were grown in a controlled environment at 200 μmol m^–^^2^ s^–^^1^ photosynthetic photon flux density [PPFD; White LED (5700 K) illumination with supplementary FAR LEDs; PSI, Drásov, Czech Republic], with 12/12-h light/dark period, 24/22 ℃ day/night temperatures and a relative humidity of 55%–60% for at least five weeks. 7- to 8-week-old intact plants were used for the experiments in 8–9 developed leaf level stages. Leaves of tomato plants were treated foliar using a squirrel hair brush with CHT experimental solution on the 6^th^ leaf level. Firstly, stock solution was prepared, CHT (Sigma-Aldrich, St. Louis, MO, USA) was dissolved in 100 mM acetic acid (AA) buffer pH 3.6 [63] to a concentration of 10 mg mL^–^^1^. From this stock solution then an experimental solution was made containing 100 μg mL^–^^1^ CHT, 1 mM AA, 10 mM 2-(N-morpholino) ethanesulfonic acid (MES), and 10 mM KCl (pH 6.15). As a control an experimental solution without CHT was used [36]. 

### 4.2. Treatments

**Figure 11:**
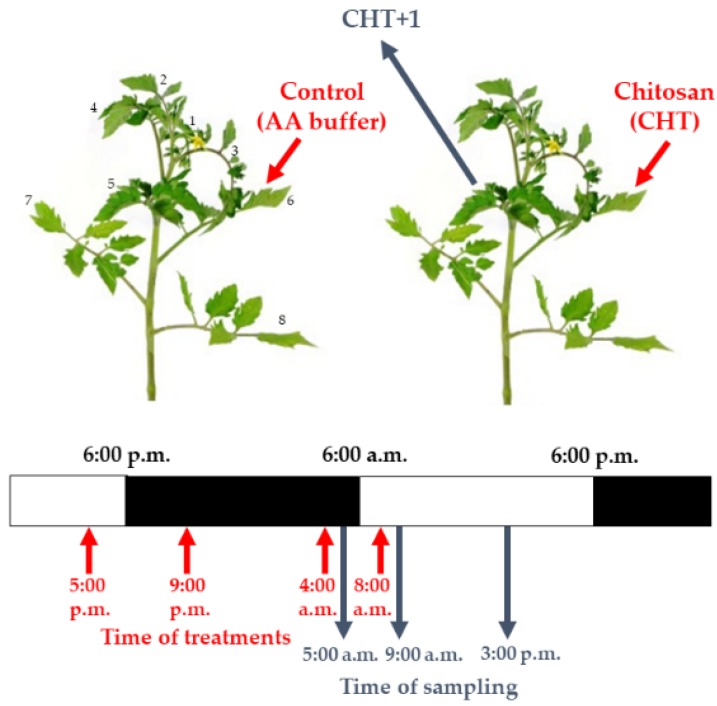
Experimental setup of chitosan (CHT) treatments and time of samplings.

To study the daytime- and light-dependent effects of CHT, we treated leaves on the 6th leaf levels of intact tomato plants at several daytimes, i.e. in the late afternoon (17:00 hrs), in the evening (21:00 hrs), at dawn (04:00 hrs) and in the morning (08:00 hrs) and we measured the plant defence responses at different time points (05:00; 09:00, and 15:00 hrs) after each treatment (except after the treatment in the morning). To examine whether light regulation plays role in CHT-induced defence reactions, artificial darkening experiments were set up in the morning (from 08:00 hrs) and the effect of CHT was similarly detected during the day at 09:00 and at 15:00 hrs. The applied day/night cycle consisted of 12 h light (200 µmol m^–^^2^ s^–^^1^ photon flux density) starting from 06:00 until 18:00 hrs and 12 h dark period during the remaining daytime. To detect the role of the first line of defence, the CHT-treated 6th leaf levels from the shoot apex were used for the experiments. To detect the systemic responses of tomato plants, the distal 5th leaf levels above the CHT-treated ones were analyzed (Figure 11).

### 4.3. Preparation of epidermal strips

Epidermal strips were prepared manually from the abaxial surface of CHT-treated (6th leaf level from the shoot apex) and distal (5th leaf level from the shoot apex) tomato leaves with forceps immediately at the sampling time. Then strips were transferred to 35 mm diameter glass bottom culture dishes (MatTek Co., Ashland, MA, USA) containing 3.5 mL of incubation buffer (10 mM MES and 10 mM KCl, pH 6.15 with TRIS) as described by Zhang et al. [64]. Strips were fixed to the bottom of the dishes with a metal grid (1 cm^2^) and were used immediately for the experiments.

### 4.4. Stomatal Aperture Measurements

At various time points, the epidermis of leaves was peeled off and immediately observed under a microscope (Nikon Eclipse TS-100, Nikon Instruments, Tokyo, Japan) as earlier described by Melotto et al. [65]. The width of stomatal pores was measured from randomly chosen areas of the pictures by Image-Pro Plus 5.1 software (Media Cybernetics, Inc., Rockville, MD, USA). 30–40 stomata of three different plants per experiments from three independent experiments were recorded. 

### 4.5. Determination of ROS and NO Production of Stomata 

Production of ROS and NO in stomata were detected using 2,7-dichlorodihydrofluorescein diacetate (H_2_DC-FDA) (Sigma-Aldrich, St. Louis, MO, USA) for ROS [66] and 4-amino-5-methylamino-2’,7’-difluorofluorescein diacetate (DAF-FM DA) (Sigma-Aldrich, St. Louis, MO, USA) for NO staining [67]. Epidermal peels were loaded with 10 μM H_2_DC-FDA or DAF-FM DA for 30 min in the incubation buffer (10 mM MES, 10 mM KCl, pH 6.15 with TRIS) in the dark at room temperature. The excess fluorophore was rinsed out twice with the incubation buffer. 

Fluorescence intensity was determined by Zeiss Axiowert 200 M type fluorescence microscope (Carl Zeiss Inc., Jena, Germany). Digital images were taken from the strips with a high-resolution digital camera (Axiocam HR, HQ CCD camera) using a filter set 10 (excitation: 450–495 nm, emission: 515-565 nm) and the fluorescence intensity was measured with Axiovision Rel. 4.8 (Carl Zeiss Inc., Munich, Germany) software. At least 10 stomata of three different plants per experiments from three independent experiments were recorded. 

### 4.6. Measurement of Photosynthetic Activity

Chlorophyll *a* fluorescence in intact leaves of tomato plants was measured with pulse amplitude modulation (PAM) chlorophyll fluorometer (PAM-2000; Heinz-Walz, Effeltrich, Germany). Leaves were dark-adapted for 15 min before the measurements as previously described by Poór and Tari [48]. In each treatment, three independent leaves were measured. 

Chlorophyll *a* fluorescence was also measured in stomata by a specific PAM chlorophyll fluorometer (Microscopy-PAM; Heinz Waltz GmbH, Germany) mounted on a Zeiss Axiovert 40 inverted epifluorescence microscope (Carl Zeiss Inc., Jena, Germany) under the same experimental conditions at room temperature in the dark. One or two stomata (free from adhered mesophyll cells) were measured at random in three different epidermal strips from different plants in each treatment. Epidermal strips were immediately incubated after the peeling for 10 min in the dark under the microscope as dark adaptation. 

The following chlorophyll *a* fluorescence parameters were calculated in each experiments: the maximal quantum efficiency of photosystem II (PSII) photochemistry: F_v_/F_m_ = (F_m_ - F_0_)/F_m_; the actual quantum yield of PSII electron transport in the light-adapted state: Φ_PSII_ = (F_m_’ - F_s_)/F_m_’; the photochemical quenching coefficient, qP = (F_m_’ - F_s_)/(F_m_’ - F_0_’), the light induced photoprotection through thermal dissipation of energy: NPQ = (F_m_ - F_m_’)/ F_m_’ [68,69].

### 4.7. RNA Extraction, Expression Analyses by Quantitative Real-time PCR

Quantitative real-time reverse transcription-PCR (qRT-PCR; qTOWER Real-Time qPCR System, Analytik Jena, Jena, Germany) was used to detect the expression pattern of the selected tomato genes (*SlPR1* (Solyc01g106620): F: 5’-CATCCCGAGCACAAAACTATG-3’, R: 5’-CCCCAGCACCAGAATGAAT-3’; *SlERF1* (Solyc05g051200): F: 5’-GGAACATTTGATACTGCTGAAGA-3’, R: 5’-AGAGACCAAGGACCCCTCAT-3’) mined from Sol Genomics Network (SGN; http://solgenomics.net/) database [70]. The PCR reaction mixture contained 10 ng cDNA template, 400–400 nM forward and reverse primers, 5 µL of Maxima SYBR Green qPCR Master Mix (2X) (Thermo Scientific, Waltham, MA, USA) and nuclease-free water (denaturation at 95 ℃ for 7 min, followed by 40 cycles of denaturation at 95 ℃ for 15 s and annealing extension at 60 ℃ for 60 s) in 10 L volume. qTOWER Software 2.2 (Analytik Jena, Jena, Germany) was used to analyse the data. Tomato 18S rRNA and elongation factor-1α subunit were used as reference genes and the expression data were calculated by the 2^(^^–^^∆∆Ct)^ formula [71]. Data were normalized to the transcript levels of tomato reference genes, and to the transcript levels of untreated control leaves in each time point. 

### 4.8. Statistical Analysis

Experiments were repeated at least two times. The results are expressed as means ± S.E. The effects of CHT treatments and the differences among the daytimes were analyzed by two-way ANOVA, supplemented with post hoc pairwise comparisons using the Holm-Sidak test. All statistical analysis was performed using SigmaPlot 11 software (Systat Software Inc., San Jose, California, USA). Mean values denoted with different letters differed significantly at *P* < 0.05. 

## 5. Conclusions

It can be concluded that CHT has a daytime- and light-dependent effects on intact tomato plants because it caused stomatal closure before dark/light transition only in leaves treated at 17:00 hrs but not in night-treated ones. However, it also induced stomatal closure at 09:00 hrs in plants treated at dawn and in the morning. At the same time, CHT induced the generation of ROS in guard cells in the first part of the light phase independently of the daytime of the application. ΦPSII decreased in guard cells upon CHT treatments at 09:00 treated at 17:00 hrs, which together with higher ROS production can contribute to the stomatal closure at this time point. Higher expression of *PR1* was already detected at dawn but it reached the maximum at 09:00 hrs after the CHT treatment at night suggesting a delayed and daytime-dependent defence response of tomato plants. At the same time, CHT-mediated changes are also worthy of further investigation in mutant model plants of the circadian clock elements. However, not only the daytime- but light-dependent regulation of CHT-induced defence response was also confirmed with artificial dark treatment because CHT-induced *PR1* expression was inhibited by the dark. CHT-induced systemic response was also observed for the first time. In the distal leaves of CHT-treated ones, significant stomatal closure was measured in the morning. In these leaves, high ROS/NO production and elevated expression of *PR1* and *ERF1* were also detected, confirming that CHT can activate SAR within the first hour in the light phase of the day in intact tomato plants. These observed physiological and molecular changes in tomato leaves can highlight the importance of the daytime and light in the early responses of intact plants to fungal elicitor treatments.
